# Local Interaction Density (LID), a Fast and Efficient Tool to Prioritize Docking Poses

**DOI:** 10.3390/molecules24142610

**Published:** 2019-07-18

**Authors:** Célien Jacquemard, Viet-Khoa Tran-Nguyen, Malgorzata N. Drwal, Didier Rognan, Esther Kellenberger

**Affiliations:** Laboratoire D’innovation Thérapeutique, UMR7200, CNRS, Université de Strasbourg, 67400 Illkirch, France

**Keywords:** scoring, protein ligand interaction, benchmarking

## Abstract

Ligand docking at a protein site can be improved by prioritizing poses by similarity to validated binding modes found in the crystal structures of ligand/protein complexes. The interactions formed in the predicted model are searched in each of the reference 3D structures, taken individually. We propose to merge the information provided by all references, creating a single representation of all known binding modes. The method is called LID, an acronym for Local Interaction Density. LID was benchmarked in a pose prediction exercise on 19 proteins and 1382 ligands using PLANTS as docking software. It was also tested in a virtual screening challenge on eight proteins, with a dataset of 140,000 compounds from DUD-E and PubChem. LID significantly improved the performance of the docking program in both pose prediction and virtual screening. The gain is comparable to that obtained with a rescoring approach based on the individual comparison of reference binding modes (the GRIM method). Importantly, LID is effective with a small number of references. LID calculation time is negligible compared to the docking time.

## 1. Introduction

Predicting how a ligand binds to a protein is one of the challenges of structural bioinformatics. In the early eighties, Kuntz et al. proposed a geometric model of molecular recognition [1]. Since then, a plethora of programs have been developed to dock a ligand at its protein site based on both shape and electrostatic (or pharmacophoric) complementary. The protein is mostly treated as rigid body and ligand conformations are sampled by varying torsion angles. This approach allows for rapid prediction, so that docking has established itself as a method of choice for high throughput applications such as virtual screening. The literature reports many cases of identification of bioactive compounds by serial docking to a target protein [2]. However, examples confirming experimentally the predicted poses are rarer [3]. Benchmarking studies show that the quality of prediction is variable, although significant progress has been made over the past 15 years. The weakness of docking lies mainly in the scoring functions [4,5]. The widely used docking programs are generally able to predict a ligand/protein three-dimensional (3D) structure similar to that observed by X-ray crystallography, but their scoring function does not necessarily reward it as the best. Logically, scoring functions are not very effective in more difficult exercises, such as distinguishing between active and inactive molecules on a target protein and ranking active molecules by binding affinity [6,7]. A recent review by Guedes et al. provides a good overview of empirical functions and their recent and future developments, while discussing the evolution in the design of test datasets, the contribution of learning machines and challenging topics [8].

Docking performance in pose prediction and virtual screening can be improved by post-processing the docking poses based on the analysis of the interactions formed between the ligand and the protein. The underlying assumption is that the binding mode of a relevant pose shares similarities with experimentally validated binding modes. The suggestion of Deng et al. in 2004 to convert interactions in a numerical fingerprint naturally led to the design of several simple and fast methods to compare two binding modes [9]. Similarity is evaluated according to the presence/absence of interactions in the two interaction fingerprints [10]. Interaction fingerprint has become a useful tool for drug discovery [11]. In 2013, we proposed to encode the binding mode by an interaction pattern graph, so that the similarity score also takes into account the spatial relationships of interacting atoms. This method, called GRIM, is overall more efficient than our in-house interaction fingerprint (IFP) in pose prediction and in virtual screening, but is also more costly in computation time [12,13]. The advantages of GRIM rescoring with respect to standard energy-based scoring functions have notably been acknowledged for various targets in two recent international docking contests [14,15].

**Figure 1:**
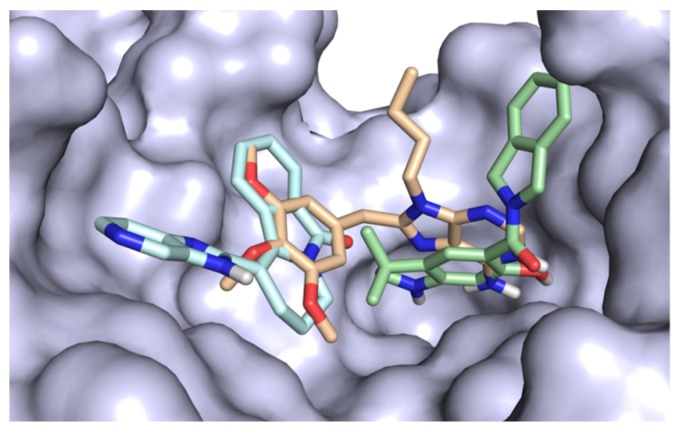
Different binding modes to the heat shock protein HSP 90-alpha. The protein surface is colored in grey, the ligand carbon atoms in cyan (HET code: YJX, PDB code: 2YJX), light orange (HET code: PU3, PDB code: 1UY6) and pale green (HET code: L81, PDB code: 2YJX).

The success of rescoring with GRIM or IFP depends on the experimental reference 3D structures, which must include a relevant binding mode (Figure 1). In addition, the approach is not applicable to proteins that have not been crystallized in the presence of a drug-like molecule, thus neglecting all the information provided by ions, solvent molecules or any other additive present in the binding site [16]. In this study, we propose to combine the multiple experimental binding modes into a single reference, in order to make the rescoring approach more robust, faster and applicable to a larger number of cases.

In this article, we describe the method, called Local Interaction Density or LID. LID and GRIM being based on the same representation of the binding mode, LID’s performance in pose prediction and virtual screening are compared to that of GRIM.

The pose prediction was based on a high quality dataset [17]. Each protein was described by at least 20 3D structures containing diverse drug-like ligands. This allowed us to quantify the minimum number of 3D structures needed to create a single useful reference. For three of the proteins, free protein 3D structures with crystallization additives in the binding site were available. We have therefore assessed whether crystallization additives alone are sufficient to create a useful reference.

Virtual screening was performed on eight target sets of the DUD-E dataset [18] meeting the LID requirements, i.e., those described by several experimental reference 3D structures. For one of the proteins, we also performed a virtual screening on a more challenging dataset which was created from the results of an experimental screening [19].

## 2. Results and Discussion

### 2.1. Description of the LID Method

The LID method consists of two steps: (i) the creation of maps describing all the binding modes of a protein, and (ii) the calculation of a score for posing a ligand.

#### 2.1.1. Creation of LID Maps

**Figure 2:**
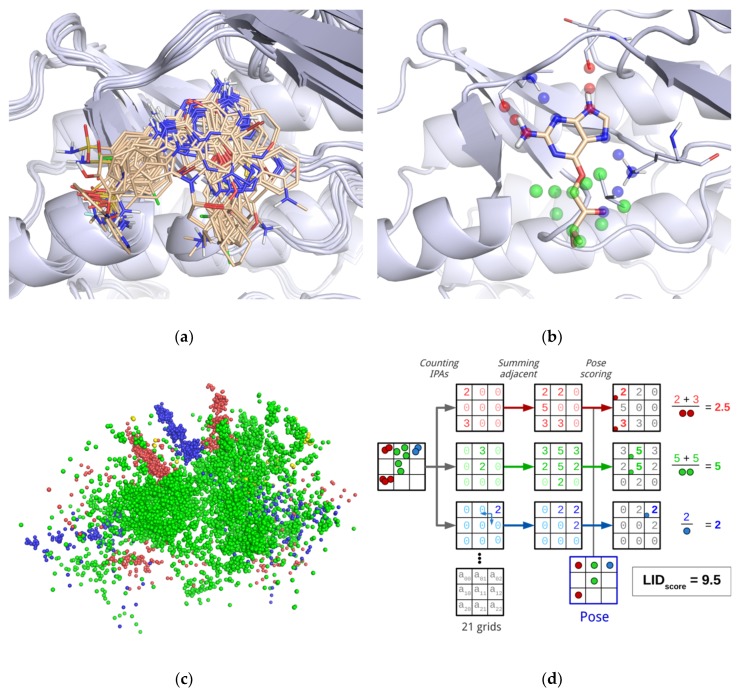
LID method. (**a**) superposition of the reference 3D structures onto the representative 3D structure of the protein binding site; example of CDK2 (PDB code: 2B53); (**b**) detection of interactions between the protein and its ligand. Hydrogen bonds (blue and red interaction pseudo-atoms) and hydrophobic contacts (green interaction pseudo-atoms) between CDK2 and its ligand (PDB code: 1GZ8, HET code: MBP); (**c**) ensemble of superimposed interaction pseudo-atoms; (**d**) LID maps: generation from the merged interaction pseudo-atoms and used in rescoring. For the sake of clarity, only 3 of the 21 maps are represented. In (**a**) and (**b**), proteins are represented by light grey ribbons and ligands by sticks (carbon atoms in pale orange). In (**a**), (**b**) and (**c**), interaction pseudo-atoms are represented by spheres, colored according to the corresponding bond type. The images in (**a**), (**b**) and (**c**) show the same view.

All protein 3D structures were superposed onto a single 3D structure whose binding site was representative of the structural ensemble (Figure 2a). In each 3D structure, non-covalent interactions were detected using IChem [20]. We considered five interaction types: hydrogen bond (HB), ionic bond, metal chelation, π-stacking and hydrophobic contacts. In addition, IChem distinguished HB donor and HB acceptor subtypes, whether the donor was a ligand or a protein atom. Similarly, it distinguished cationic or anionic subtypes of ionic bond whether the cation was a ligand or a protein atom. In total, there were seven IChem interaction types. An interaction was represented by a triplet of interaction pseudo-atoms (IPA) (Figure 2b), the first was positioned on the ligand atom (IPA_ligand_), the second on the protein atom (IPA_protein_) and the third at mid-distance between the first two (IPA_center_) (Figure 2b). Consequently, there were 21 pseudo-atom types (7 interaction types × 3 position tags).

The triplets of IPAs generated from all reference 3D structures were then fused (Figure 2c), and placed in a cubic grid with an edge length equal to 0.1 Å. The grid was built in the frame of the representative reference 3D structure. The grid boundaries were fixed by the two furthest IPAs which represented the diagonal and defined the center of voxels. To avoid edge effects, each voxel was assigned a density score which was the sum of the number of IPAs it contained and the number of IPAs contained in the adjacent voxel based on a Manhattan distance of 0.5 Å (Figure 2d). Each IPA type was considered separately, yielding 21 maps.

#### 2.1.2. Calculation of the LID Score

The LID score was obtained by comparing the docking pose IPAs with the LID maps (Figure 2d). For each IPA type, i.e., for each of the 21 LID maps, the density scores were summed across all voxels and the resulting sum was divided by the number of docking pose IPAs. The LID score was the sum of individual scores calculated from the 21 maps. A high LID score means that a high proportion of the docking pose interactions are found in the reference complexes and that the docking pose interactions are observed in a large number of reference complexes. The calculation of the LID score is formalized by the following equation:

(1) $$LID_{score}=\sum_{i=1}^{N_{IPA}}\frac{1}{N\left(M_{i},T_{i}\right)}G\left(x_{i},y_{i},z_{i},M_{i},T_{i}\right),$$

where *G* represents the interaction grid, *x_i_*, *y_i_*, and *z_i_* are the three cartesian coordinates of IPA*_i_*, *M_i_* is the IPA*_i_* mode (i.e., IPA_ligand_, IPA_center_ or IPA_protein_), *T_i_* is the IPA*_i_* interaction type (e.g., HB) and *N*(*M_i_*,*T_i_*) is the number of IPAs with the same mode and type.

For comparison, the GRIM score quantifies the similarity between two IPA patterns, that of the docking pose and that of a reference structure. It was empirically determined by fitting six parameters to a shape-based similarity score on 1800 pairs of protein–ligand complexes (900 similar and 900 dissimilar) [12]. The six parameters take into account: the number of matched IPA_ligand_; the number of matched IPA_center_; the number of matched IPA_protein_; the proportion of matched IPAs weighted by interaction type; the quality of the superimposition of matched IPAs; and the difference in the total number of IPAs between the docked pose and the reference pose.

The LID and GRIM scores are both additive scores. However, the LID score is obtained exclusively from positive contributions, i.e., elements common to the binding modes compared, while the GRIM score penalizes differences in geometry and the size of the compared binding modes. In addition, unlike the GRIM score, the LID score does not weight the contribution of interactions according to their types. Finally, the LID score, being determined by a set of 3D reference structures, is therefore customized for a particular site. On the contrary, the GRIM score was designed to be universal.

### 2.2. LID’s Performance in Pose Prediction

Is LID able to recognize, among the docking poses selected by the scoring function, those that are close to the crystallographic structure of the ligand-protein complex? To answer this question, we sought to reproduce the crystallographic structures of 1382 ligand-protein complexes. These 3D structures have been carefully selected in the Protein Data Bank (PDB) to meet strict quality criteria (e.g., no mutation in the site, agreement between atomic coordinates and electron density), while being globally adapted to the docking approach (drug-like ligand, ligandable site).

The test dataset, called the LID dataset, describes 19 proteins (Table S1). On average, a protein is described by about fifty different 3D structures. There are almost as many different ligands in complex with a protein as there are 3D structures of that protein (there can be more than one structure of the same complex). Four of the proteins are represented by more than 100 3D structures: cyclin-dependent kinase 2 (CDK2) with 156 ligands, carbonic anhydrase 2 (CAH2) with 155 ligands, beta-secretase 1 (BACE1) with 152 ligands, and heat shock protein 90-alpha (HSP90A) with 106 ligands. Table 1 gives the number of ligands per protein. Protein descriptors indicate whether the binding site is rather large or small, hydrophilic or hydrophobic, rigid or flexible. All cases are encountered in the dataset. In four proteins, the binding site contains a metal cation. The 19 proteins exhibit a variable proportion of interaction types with bound ligands (Table S2).

The evaluation of the LID rescoring approach in pose prediction was performed as follows. The ligands’ input 3D structures were generated from their SMILES codes. Up to 20 3D structures were obtained for the same SMILES code if several conformations were possible for a cyclic compound (e.g., cyclohexane in the chair or the boat conformation, substituent in the axial or the equatorial position). All the ligands of a protein were docked into the same site, using all 3D structures of the protein, except that of the self crystallographic complex. In other words, we did non-native or cross-docking. For each docking job, the 10 poses with the highest docking scores were evaluated with LID. Up to 45,200 poses were compared for the same complex. The docking poses were also evaluated with GRIM.

**Figure 3:**
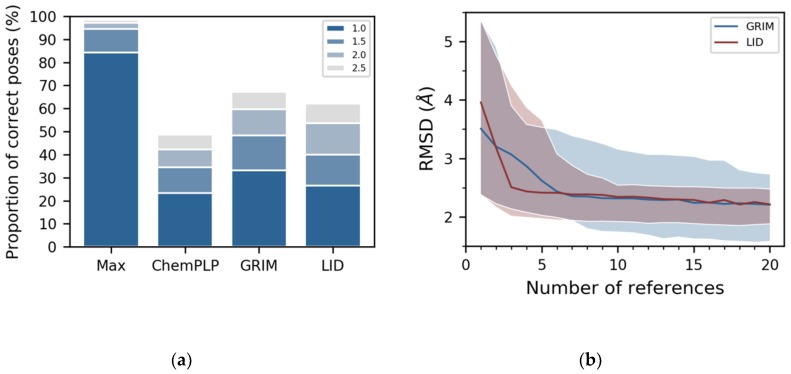
LID’s performance in pose prediction. (**a**) comparison with GRIM and ChemPLP. The legend indicates the threshold in Å under which poses are considered as correct. “Max” represents the proportion of correct poses according to the best possible RMSD criterion; (**b**) as a function of the number of reference 3D structures used to build the map. The distributions of median RMSDs obtained for the 19 proteins in the dataset using LID and GRIM are shown in red and blue, respectively. A line is drawn at the median value calculated on the 19 proteins, and the colored area delimits the first and ninth deciles of the distribution.

The docking was carried out with the PLANTS software, which has the advantage of being freely distributed, and which, in our hands, reproduces the diversity of poses obtained with the GOLD program (using “enable the generate diverse solutions” and disabling “allow early termination”) [21]. Tested on the LID dataset, PLANTS found a pose close to the crystallographic structure in 1313 out of the 1382 complexes (Figure 3a, see “Max” labelled bar). Here, we evaluated the similarity between the docked pose and the crystallographic structure using the RMSD calculated on the non-hydrogen atoms of the ligand, and we considered that the docked pose is correct whether the RMSD is below 2 Å. The PLANTS default scoring function, namely ChemPLP, placed a correct pose in the first position in only 42% of the cases (Figure 3a, see “ChemPLP” labelled bar), while GRIM did so in 60% (Figure 3a, see “GRIM” labelled bar). LID score performed almost as well as GRIM, placing at the top position a correct pose in 54% of cases (Figure 3a, see “LID” labelled bar). In particular, LID is less effective than GRIM in the selection of ligand poses for four proteins: the phosphodiesterase 10A (PDE10), the protein kinase Chk1 (CHK1), the epoxide hydrolase 2 (HYES) and the leukotriene A-4 hydrolase (LKHA4) (Table S3).

### 2.3. LID’s Advantages and Limitations

As mentioned above, GRIM and LID are both based on IChem tools for the detection of ligand-protein interactions, and their encoding in IPAs. They, however, differ in two aspects: firstly, GRIM comparison of binding modes is based on clique detection and therefore does not require the pre-alignment of the compared 3D structures, unlike LID; secondly, GRIM rescoring considers the reference 3D structures individually, while LID considers the reference 3D structures as a whole. As a consequence, we expect LID to be less sensitive than GRIM to the number of reference 3D structures, but more sensitive to structural variations in protein coordinates.

We assessed the extent to which GRIM and LID depended on the number of reference 3D structures by repeating the rescoring with a growing number of reference 3D structures. For each protein in the dataset, we tested all combinations of *n* reference 3D structures, with *n* ranging from 1 to 20 (the maximal number of tested combinations is equal to 1000). We thus generated up to 1000 × 21 new LID maps per protein. GRIM and LID best performance was observed for 10 or more reference 3D structures (Figure 3b). The decrease of the median RMSD however differed between GRIM and LID. GRIM curve shows a regular downward slope while LID curve shows a steep initial slope that becomes more gradual from three reference 3D structures on (Figure 3b). This suggests that the binding modes of three randomly chosen ligands may provide sufficient information to guide docking using LID. Moreover, from seven reference 3D structures on, LID achieves its best performance level for the majority of the tested cases. For comparison, GRIM showed larger deviations, even considering a larger number of reference 3D structures.

### 2.4. Application of LID to “apo” Proteins

**Figure 4:**
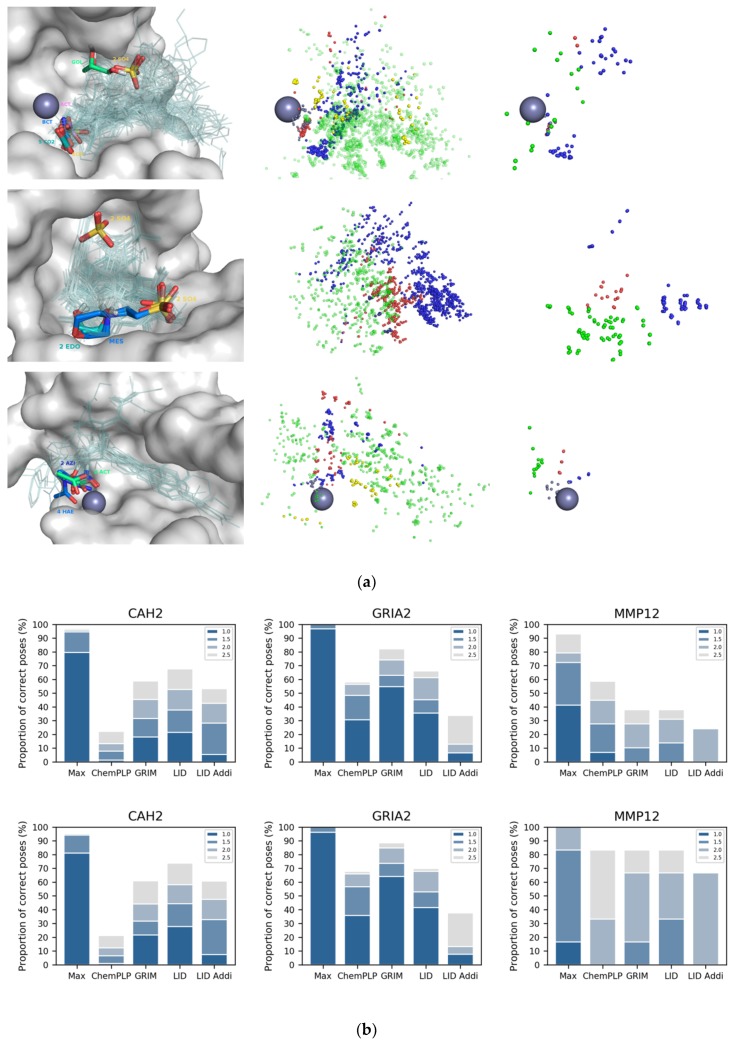
Use of crystallization additives binding modes in LID. (**a**) additives crystallized in CAH2 (top), GRIA2 (middle) and MMP12 (bottom). Metal cation is represented with a grey sphere. On the left triad are shown the additives (thick sticks colored by HET code) and drug-like ligands (transparent lines) in the protein site (grey surface); on the middle triad are shown the drug-like ligands interaction pseudo-atoms, colored according to the corresponding bond; on the right triad are shown the additives interaction pseudo-atoms, colored according to the corresponding bond type (hydrogen bond in red and blue, π-stacking in yellow, hydrophobic contact in green and metal chelation in grey); (**b**) LID’s performance in pose prediction of drug-like ligand (top) and fragment only (bottom). LID and LID Addi refer to the use of drug-like ligands and additives as reference, respectively.

LID and GRIM have been designed for already well characterized protein structures, for which binding mode information is available for at least one ligand. Is it possible to use LID for a protein whose structure has been resolved at the atomic level, in the absence of drug-like ligands but in the presence of diverse crystallization additives? To answer this question, we searched the LID dataset for proteins that are represented in the PDB in the form “apo” (i.e., in the absence of drug-like ligand) but whose site is not empty. Three proteins have at least three different additives in their binding site: CAH2, macrophage metalloelastase (MMP12) and glutamate receptor (GRIA2) (Figure 4a). Glycerol, sulfate, acetate, carbon dioxide, bicarbonate or cyanic acid are found in CAH2 (carbon dioxide and bicarbonate are ligands of the enzyme that have a key role in regulating cell pH). Acetic and acetohydroxamic acids and an azide ion are found in MMP12. Sulphate, ethanediol and morpholinoethanesulphonic acid are found in GRIA2. Both CAH2 and MMP2 are metalloproteins, and in the 3D structures of the two proteins, we observed at least one anionic additive being a coordinating ligand of the metal cation (see the grey IPAs on Figure 4a). New LID maps were built using only the interactions detected between proteins and additives (note that in the case of CAH2 and MMP12, the three LID maps corresponding to ligand–metal interactions contain information). They yielded the identification of a correct pose for nearly half of the 155 CAH2 ligands (Figure 4b). For comparison, ChemPLP is three times less efficient. Opposite results were observed for GRIA2 and MMP12. We suspected the approach not to be suitable for large ligands, and therefore did the analysis again for fragments only (i.e., drug-like ligand with MW ≤ 300, number of non-hydrogen atoms ≤ 18). We confirmed that additives effectively helped to predict ligand placement in CAH2 and MMP12, but not in GRIA2.

Is it possible to predict whether the information provided by the additives alone is relevant for rescoring with LID? Distribution of IPAs suggested a positive answer. In the three study cases, additives revealed a motif of directional interactions, which was conserved in the ligands’ binding modes (Figure 4a). However, the amino acids involved in these interactions were mostly rigid in CAH2 and MMP12 (all atom RMSD ~ 0.5 Å) while they adopted different conformations in GRIA2 (all atom RMSD ~ 1.7 Å). In summary, the LID approach using additives can be considered for fragment docking if the protein site is rigid. Since flexibility is not necessarily revealed by the 3D structures of “apo” proteins, it is nevertheless advisable to consider crystallographic structural factors or to perform a molecular dynamics simulation [22,23].

### 2.5. LID’s Performance in Virtual Screening

It is observed that ligand pose prediction performance by molecular docking was already improved with LID. At this point, another question arises as to whether LID is also capable of efficiently discriminating between true active compounds of a given protein and their chemically similar decoys or not. To answer this question, a retrospective virtual screening challenge using a set of DUD-E targets was carried out, with LID employed as docking-assistant tool. Only eight protein targets included in both the DUD-E and the LID datasets as described above were investigated. These include: the aldo-keto reductase (ALDR), BACE1, CAH2, CDK2, the estrogen receptor alpha (ESR1), GRIA2, HSP90A, and LKHA4 (Table 1, Table S1). A rigid docking protocol using a unique representative 3D structure for each target’s binding site was implemented. True actives and decoys were already prepared by the DUD-E contributors and used as such. Each ligand corresponded to a maximum of eight stereoisomers, each of which issued 10 post-docking poses that were kept and analyzed.

**Figure 5:**
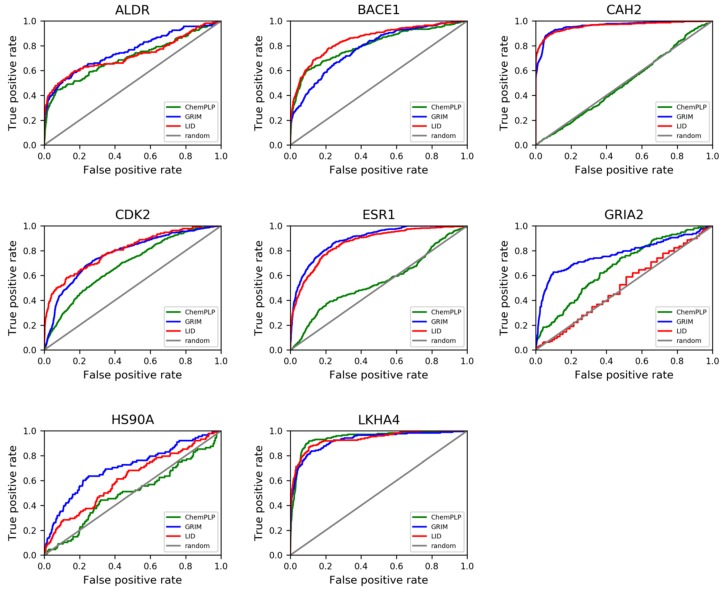
LID’s performances in retrospective virtual screening. All of the ROC curves show screening results with DUD-E. LID’s performances are compared to those of GRIM and ChemPLP.

LID’s performance was evaluated according to the Receiver Operating Characteristic (ROC) curves that were obtained (Figure 5). It is observed that LID generally gave better performances than ChemPLP, with a mean area under the ROC curves (ROC AUCs) of 0.78, compared to an average value of 0.67 issued from the latter method (Table S4). The enrichment factors were also significantly improved except for GRIA2 (Table 1). On the whole, the improvement brought by LID was quantitatively comparable to that by GRIM. However, the rescoring with LID took up a remarkably shorter amount of time (Table 2). This observation became more obvious when a docking process using multiple structures of a single protein was carried out. To be more specific, 10 structures were used as input for each of the two most flexible protein targets HS90A and GRIA2, with the aim of taking into account the most diverse structures of the ligand-protein binding site. A docking-based virtual screening process was conducted for each structure. The average recorded calculation time for GRIM rescoring was approximately 58 h per protein structure, while that with LID was only 10 min (noteworthy, GRIM was coded in C++, while LID was coded with Python). Nevertheless, the time required to align all protein structures for LID to function properly has to be considered. In terms of overall performances, the multitarget approach using LID was observed to give notably better results in HS90A and GRIA2, with an improvement of 9.30 and 19.0 in true positive percent at 5% decoys, respectively (Table S5).

The DUD-E datasets have long been employed for a benchmarking of novel structure-based methods in computer-aided drug design. However, the real value of these datasets has been subject to much debate, primarily due to serious drawbacks in compound selection, e.g., the structural biases that led to artificial enrichment and an overestimation of virtual screening performance, or the fact that the potency of the decoys always remained unknown [24]. We therefore put LID into another retrospective virtual screening challenge using a dataset whose active and inactive compounds along with their potency were already verified by confirmatory dose-response biological assays. This dataset, ESR1, was prepared from the results of a high-throughput screening of small molecule antagonists of the estrogen receptor alpha that can be accessed on the website of PubChem BioAssays (for more details concerning the compound selection, see [19]). The dataset employed in this study comprises 1589 compounds, 59 of which are actives with potency values ranging from 3.9 nM to 9.6984 µM; the rest (1530 compounds) were confirmed as inactives.

**Figure 6:**
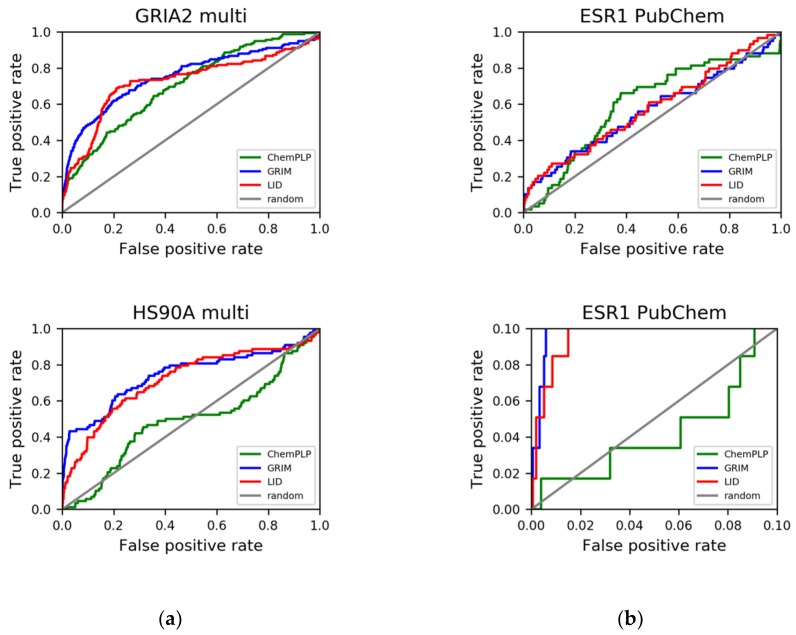
LID’s performances in retrospective virtual screening using 10 protein structures. (**a**) virtual screening with the DUD-E dataset; (**b**) virtual screening with the cleaned PubChem Bioassay dataset ESR1 (AID 743080). The bottom is an enlargement of the full ROC curve. LID’s performances are compared to those of GRIM and ChemPLP.

As already observed with DUD-E, the ChemPLP scoring function gave an extremely poor performance in the screening challenge with ESR1. LID and GRIM did not give good performances like in the cases of DUD-E datasets (the two approaches had more difficulty in distinguishing between true actives and true inactives than in separating true actives from their chemically similar decoys); however, both methods were capable of selecting true actives among the early hits, with 18.6% and 16.9% of active compounds retrieved at a constant false positive rate of 5%, respectively (Figure 6b).

### 2.6. LID Cost in Calculation Time

The LID method has been designed to process a very large number of poses, typically in a virtual screening of large libraries using multiple 3D structures of the target protein. The tests performed as part of this study allowed us to estimate the relative time required for such an application (Table 2, Table S6). The generation of LID maps, whose quantity is proportional to the number of reference 3D structures (N_ref_), was made once. The generation time on a desktop computer was about 0.18 × N_ref_ seconds (R^2^ = 0.8). LID score calculation lasted ~0.003 s per pose (R^2^ = 0.6) with an Intel Xeon E3-1240 (3.70 GHz) processor. LID score calculation better correlated with the number of compared IPAs (R^2^ > 0.95), yet this information is not known before rescoring. Nevertheless, the first approximation is valid since rescoring total time with LID is negligible compared to docking time. For the sake of comparison, GRIM rescoring time was longer and not proportional to the number of docked poses or reference 3D structures. Due to its clique detection step, GRIM calculation time highly depended on the number, types and distribution of IPAs (Table S7). The difference between LID and GRIM calculation times in seconds was up to several orders of magnitude.

### 2.7. Comparison with Other Methods Using Aligned 3D Structures

The first strategies for using crystallographic structures of ligand-protein complexes in docking methods are almost 20 years old [25,26]. Different ways of incorporating structural information have been proposed, for example by introducing spatial constraints targeting parts of the ligand that are common with a reference ligand, by guiding the placement of the ligand on reference atom-centered functions or electron density, or by scoring pose by 3D pharmacophore matching similarity [27,28,29]. Okuno et al. suggested to combine multiple ligand-protein crystallographic structure into a reference grid [30,31]. The method, called VS-APPLE, aligned the ligand with the reference grid to predict the binding mode. Designed as a screening method, it was able to discriminate active compounds from decoys of the DUD dataset [32]. The LID and VS-APPLE methods are similar. However, LID is intended exclusively for scoring purposes and is not linked to a particular docking program. LID especially allows to take into account induced fit using a docking program that treats the protein as a rigid body. The method is indeed fast and efficient enough to sort the multiple poses of a ligand docked into an ensemble of 3D structures of its protein site. LID’s performance assessment also indicated that the method can be successful using a limited amount of reference information. We estimated that less than ten different ligands are required for effective scoring. This is in line with our recent analysis of binding modes in PDB, which concluded that nine ligands achieve the coverage of interactions formed with the protein pocket [17]. Similarly, VS-APPLE good performance was also reached with an incomplete reference grid. The minimum information required on the 13 studied proteins of the DUD dataset was estimated at 30 percent of the total atomic coordinates.

## 3. Materials and Methods

### 3.1. Dataset Preparation

#### 3.1.1. Pose Prediction Challenge

The LID dataset was prepared as previously described [17]. The 3D structures selected from the PDB are all in high resolution and are completely described. There is no mutation in the binding site. Drug-like ligand complies to the Lipinski’s rule of 5, with a maximum of one exception. The molecules of the LID dataset were protonated with the program Protoss v2.0 (University of Hamburg, Hamburg, Germany) [33]. Importantly, the cofactors and water molecules were removed from the protein structures.

#### 3.1.2. Retrospective Virtual Screening Challenge

Eight protein targets common to the DUD-E and the LID datasets, namely ALDR, BACE1, CAH2, CDK2, ESR1, GRIA2, HSP90A, LKHA4, were used as input and processed as described above. The representative protein structure was determined by fitting and computing RMSD matrix on Cα carbons with the command cealign in PyMOL (The PyMOL Molecular Graphics System, Version 1.8.6.1, Schrödinger, LLC, New York, NY, USA). The structure with the minimal average value was selected. For the selection of 10 structures, an agglomerative hierarchical clustering of all structures was carried out with the scikit-learn v0.19.1 Python package, then the most representative of each cluster was kept. Another dataset, ESR1, comprising true actives and true inactives confirmed by a PubChem quantitative high-throughput screening assay of small molecule antagonists of the estrogen receptor alpha was directly employed after preparation steps described by Tran-Nguyen et al. [19].

#### 3.1.3. Properties of Binding Sites

A binding site is defined as the consensus residues near the bound drug-like ligands. In each 3D structure, the protein residues with at least one non-hydrogen atom closer than 6.5 Å to any ligand non-hydrogen atom were identified. The binding site was defined as the ensemble of residues present in more than 10% of the structures [17]. The RMSD values were computed either on Cα or all non-hydrogen atoms by fitting a structure to a reference (described in LID score subsection) with the command cealign in PyMOL v1.8.6.1. Cavity volumes and the percentage of hydrophobic interactions were determined with the Volsite module in IChem v5.2.9 (UMR7200 CNRS-University of Strasbourg, Illkirch, France).

### 3.2. Docking

#### 3.2.1. Ligand Preparation

Each ligand in the SMILES file format was ionized with the program Filter of OpenEye (Filter 2.5.1.4 OpenEye Scientific Software, Santa Fe, NM, USA). The 3D structure was built with the program Corina 3.40 (Molecular Networks GmbH, Nürnberg, Germany) [34]. The option rc was used to generate multiple conformations of the rings (with a maximum of 100 stereoisomers per molecule). For CAH2 ligands with sulfonamide groups, the nitrogens linked to a sulfur atom were deprotonated (−1 charge).

#### 3.2.2. Docking with PLANTS

A rigid docking procedure was performed with the PLANTS program v1.2 using the ChemPLP scoring function and the search speed set at 1 (highest accuracy) [35].

PLANTS is based on an ant colony algorithm to optimize the placement and the conformation of ligand as well as the positions of the protein hydrogen atoms that form hydrogen bonds with the ligand. PLANTS explores possible torsion angle values of the ligand but does not modify the conformation of rings. In the case of metalloproteins, PLANTS considers geometry parameters related to metal atoms.

The cavity center of a protein site was defined from the centroid of all the ligands bound to this protein. The cavity radius was set as the maximum distance between the cavity center and the atoms of all the ligand crystallized in the binding site, plus 2 Å. On average, the radius was equal to 12 Å. Ten poses were saved per docking run.

### 3.3. Post-Processing of Docking

#### 3.3.1. GRIM Score

The GRIM score was computed with the GRIM module of the IChem program, v5.2.9, all options were kept as default [20].

#### 3.3.2. LID Score

The representative 3D structure of a protein was defined after multiple comparisons of possible binding sites using the Shaper program (minimal average distance of the matrix) (v1.0, UMR7200 CNRS-University of Strasbourg, Illkirch, France) [36]. Reference structures were aligned onto the representative with the CE program (v2003.03.13, RCSB Protein Data Bank, San Diego, CA, USA) [37]. The IPAs were obtained from the aligned reference structures with the ints module of IChem v5.2.9, the maximal distance to detect π–π stacking interactions was fixed at 5.0 Å (-D_Ar 5.0). All IPAs were merged into a mol2 file and were considered as a single pseudomolecule. This file was used for the creation of the LID maps, using the in-house intgrid program, v1.0. The LID program (v1.0) issued a LID score for each docking pose.

## 4. Conclusions

We propose LID, a novel docking-assistant tool that evaluates the relevance of protein–ligand binding modes by a comparison with those obtained from an accumulation of reference 3D structures. LID ameliorated pose prediction performance using ligands of 19 diverse proteins, and improved early enrichment of true active compounds for eight protein targets. LID’s performance was comparable to that of GRIM, a former approach based on interaction pattern graph similarity. It is noteworthy that LID was faster and more robust than GRIM if few 3D structures were available to describe a particular target. LID was in particular more adapted to high-throughput applications, e.g., high-throughput virtual screening using multiple structures of the same protein. In addition, LID allowed a consideration of binding modes of additives used during the structure crystallization process.

## Figures and Tables

**Table 1:** LID dataset description: binding site (**a**), pose prediction (**b**) and virtual screening (**c**). Proteins are sorted by the number of 3D structures in descending order (HET codes). Site HYD is the relative hydrophobicity of the binding site (ratio of polar to apolar cavity describing atoms; see Material and methods). For each descriptor/count, the nine largest values are written in bold. Median LID RMSD is the median RMSD of docked poses after LID rescoring. TP% at 5% decoys is the true positives proportion, in percent, at a constant 5% decoys rate retrieval, in a virtual screening experiment using a single protein structure.

Uniprot ID	HET Codes	RMSD ^(a)^ (Å)		N_residues_^(a)^	Site HYD ^(a)^ (%)	Metal Ions ^(a)^	Median LID RMSD ^(b)^ (Å)	TP% at 5% Decoys ^(c)^		
		Cα	All					LID	GRIM	ChemPLP
CDK2_HUMAN	**156**	**1.07**	**1.62**	**48**	**45.2**		1.87	45.5	21.3	16.7
CAH2_HUMAN	**155**	0.20	0.49	37	27.4	Zn	1.91	85.2	85.7	5.08
BACE1_HUMAN	**152**	**0.86**	**1.27**	**60**	26.2		1.79	49.5	31.5	45.2
HS90A_HUMAN	**106**	**1.45**	**1.77**	**48**	**42.8**		2.70	15.9	23.9	4.55
PIM1_HUMAN	**47**	0.31	0.63	42	**53.5**		3.21			
TNKS2_HUMAN	**39**	**0.96**	**1.62**	45	36.4		0.55			
PK3CG_HUMAN	**38**	**0.77**	**1.40**	43	**46.3**		1.76			
PDE10_HUMAN	**36**	0.28	0.46	44	34.4	Mg,Zn	2.74			
PNMT_HUMAN	**32**	0.24	0.40	42	27.4		1.98			
ESR1_HUMAN	29	0.44	0.82	**48**	**63.1**		1.72	44.9	52.2	6.79
CHK1_HUMAN	29	**0.48**	0.75	**46**	**50.4**		3.24			
HYES_HUMAN	27	**0.61**	**1.01**	**64**	**43.6**		7.32			
PDPK1_HUMAN	27	0.41	**0.94**	**49**	**47.7**		1.70			
BRD4_HUMAN	26	0.34	0.63	25	**51.8**		3.23			
ALDR_HUMAN	23	0.33	0.56	36	16.9		1.61	44.0	41.5	37.1
GRIA2_RAT	23	**1.13**	**1.27**	**51**	36.1		1.70	6.33	46.8	18.4
LKHA4_HUMAN	21	0.15	0.29	**57**	24.6	Zn	3.83	73.1	71.4	73.7
MMP12_HUMAN	21	0.23	0.46	35	36.3	Zn	3.02			
TGT_ZYMMO	20	**0.61**	**0.89**	45	37.2		0.74			

**Table 2:** Elapsed time for pose rescoring of DUD-E dataset with GRIM and LID with a single protein structure. The number of references may differ from the Table 1 because multiple copies are considered.

Uniprot ID	Poses	References	Elapsed Time (min)	
			GRIM	LID
ALDR_HUMAN	93,560	41	220	5
BACE1_HUMAN	187,060	227	3000	13
CAH2_HUMAN	325,450	156	2200	13
CDK2_HUMAN	291,260	195	2500	13
ESR1_HUMAN	214,450	37	720	17
GRIA2_RAT	123,580	62	260	4
HS90A_HUMAN	50,670	139	590	4
LKHA4_HUMAN	97,210	26	180	7

## Supplementary Materials

LID dataset is available online at http://bioinfo-pharma.u-strasbg.fr/in the fragdock archive. The LID method will be implemented in the IChem toolkit. The clean PubChem dataset will be available soon at http://bioinfo-pharma.u-strasbg.fr/. Tables S1–S7; Figure S1.
