# Supplementary material for: Local Interaction Density (LID), a Fast and Efficient Tool to Prioritize Docking Poses
Source: Molecules. 2019 Jul 18;24(14):2610. doi: 10.3390/molecules24142610 (PMC6681060; doi:10.3390/molecules24142610)
Supplement: Supplementary file 1 [file molecules-24-02610-s001.pdf]

Local Interaction Density (LID), a fast and efficient docking assistant tool

Célien Jacquemard [1], Viet-Khoa Tran-Nguyen [1], Malgorzata N. Drwal [1], Didier Rognan [1], Esther Kellenberger [1]

1 Laboratoire d'innovation thérapeutique, UMR7200, CNRS, Université de Strasbourg, 67400 Illkirch, France.

Table 1. The nineteen proteins in the LID dataset.

Uniprot ID	Name	Uniprot AC	Protein class
CDK2_HUMAN	Cyclin-dependent kinase 2	P24941	Transferase
CAH2_HUMAN	Carbonic anhydrase 2	P00918	Lyase
BACE1_HUMAN	Beta-secretase 1	P56817	Hydrolase
HS90A_HUMAN	Heat shock protein HSP 90-alpha	P07900	Chaperone
PIM1_HUMAN	Serine/threonine-protein kinase pim-1	P11309	Transferase
TNKS2_HUMAN	Poly [ADP-ribose] polymerase tankyrase-2	Q9H2K2	Transferase
PK3CG_HUMAN	Phosphatidylinositol 4,5-bisphosphate 3-kinase catalytic subunit gamma isoform	P48736	Transferase
PDE10_HUMAN	cAMP and cAMP-inhibited cGMP 3',5'-cyclic phosphodiesterase 10A	Q9Y233	Hydrolase
PNMT_HUMAN	Phenylethanolamine N-methyltransferase	P11086	Transferase
ESR1_HUMAN	Estrogen receptor	P03372	Activator
CHK1_HUMAN	Serine/threonine-protein kinase Chk1	O14757	Transferase
HYES_HUMAN	Bifunctional epoxide hydrolase 2	P34913	Hydrolase
PDPK1_HUMAN	3-phosphoinositide-dependent protein kinase 1	O15530	Transferase
BRD4_HUMAN	Bromodomain-containing protein 4	O60885	Chromatin regulator
ALDR_HUMAN	Aldo-keto reductase family 1 member B1	P15121	Oxidoreductase
GRIA2_RAT	Glutamate receptor 2	P19491	Ion channel
LKHA4_HUMAN	Leukotriene A-4 hydrolase	P09960	Hydrolase
MMP12_HUMAN	Macrophage metalloelastase	P39900	Hydrolase
TGT_ZYMMO	Queuine tRNA-ribosyltransferase	P28720	Transferase

Table 2. Proportion of interaction points generated from all structures describing the 19 proteins. (a) From a protein atom point of view. Hyd: hydrophobic contact; HBA: hydrogen-bond, acceptor on the protein; HBD: hydrogen-bond, donor on the protein.

Uniprot ID	Hyd	π-stacking	HBA[(a)]	HBD[(a)]	Ionic +[(a)]	Ionic -[(a)]	Metal	Number of points
CDK2_HUMAN	72.54	0.62	13.50	12.28	0.51	0.56	0.00	7,291
CAH2_HUMAN	64.26	2.77	8.10	14.98	0.00	0.00	9.88	4,038
BACE1_HUMAN	68.11	1.41	18.17	7.34	0.00	4.96	0.00	12,255
HS90A_HUMAN	82.38	3.22	8.60	5.40	0.26	0.14	0.00	5,720
PIM1_HUMAN	82.11	1.40	5.64	6.88	0.98	3.00	0.00	1,934
TNKS2_HUMAN	65.86	10.07	8.70	15.37	0.00	0.00	0.00	3,207
PK3CG_HUMAN	82.12	0.00	5.60	12.27	0.00	0.00	0.00	1,695
PDE10_HUMAN	79.13	10.22	1.80	8.84	0.00	0.00	0.00	1,663
PNMT_HUMAN	66.32	9.74	13.75	1.83	0.52	7.85	0.00	1,746
ESR1_HUMAN	84.95	0.27	9.33	4.53	0.00	0.93	0.00	2,252
CHK1_HUMAN	72.55	0.58	12.38	10.65	0.77	3.07	0.00	1,042
HYES_HUMAN	77.05	6.81	7.65	7.56	0.00	0.93	0.00	1,072
PDPK1_HUMAN	70.18	0.00	11.65	16.28	0.00	1.89	0.00	1,425
BRD4_HUMAN	85.02	0.56	4.40	10.02	0.00	0.00	0.00	1,068
ALDR_HUMAN	76.77	5.93	0.14	17.16	0.00	0.00	0.00	2,075
GRIA2_RAT	25.36	1.36	15.64	38.49	10.17	8.97	0.00	2,429
LKHA4_HUMAN	63.81	9.11	8.87	8.40	1.02	4.79	4.00	1,274
MMP12_HUMAN	60.98	8.40	7.29	14.67	0.00	0.00	8.65	1,179
TGT_ZYMMO	41.63	7.91	32.68	16.63	0.00	1.15	0.00	872

Table 3. Performance of pose prediction for the 19 targets

Uniprot ID	Uniprot AC	Poses median RMSD (Å)		
		**ChemPLP**	**GRIM**	**LID**
CDK2_HUMAN	P24941	2.35	1.56	1.87
CAH2_HUMAN	P00918	3.27	2.17	1.91
BACE1_HUMAN	P56817	1.76	1.41	1.79
HS90A_HUMAN	P07900	7.23	2.12	2.70
PIM1_HUMAN	P11309	4.08	3.12	3.21
TNKS2_HUMAN	Q9H2K2	0.59	0.55	0.55
PK3CG_HUMAN	P48736	1.85	1.48	1.76
PDE10_HUMAN	Q9Y233	3.97	1.35	2.74
PNMT_HUMAN	P11086	2.15	1.23	1.98
ESR1_HUMAN	P03372	0.87	1.64	1.72
CHK1_HUMAN	O14757	3.67	1.62	3.24
HYES_HUMAN	P34913	7.51	2.45	7.32
PDPK1_HUMAN	O15530	2.37	1.94	1.70
BRD4_HUMAN	O60885	4.77	2.62	3.23
ALDR_HUMAN	P15121	0.94	0.76	1.61
GRIA2_RAT	P19491	1.67	0.86	1.70
LKHA4_HUMAN	P09960	3.09	1.51	3.83
MMP12_HUMAN	P39900	2.16	2.86	3.02
TGT_ZYMMO	P28720	0.66	0.58	0.74

Table 4. AUC and logAUC of ChemPLP, LID and GRIM in virtual screening challenge with the DUD-E and PubChem (in bold) dataset. λ = 0.001.

Uniprot ID	Number of protein structures	AUC			logAUC		
		Chem PLP	GRIM	LID	ChemPLP	GRIM	LID
ALDR_HUMAN	1	70.25	75.76	72.21	35.10	43.07	44.74
BACE1_HUMAN	1	80.39	77.93	85.07	43.39	39.82	49.43
CAH2_HUMAN	1	49.61	95.96	95.89	14.06	76.69	82.63
CDK2_HUMAN	1	68.70	77.34	80.26	24.96	31.60	43.70
ESR1_HUMAN	1	55.22	88.89	86.18	17.93	47.63	47.48
	**10**	**60.02**	**56.16**	**57.72**	**18.20**	**22.49**	**22.61**
GRIA2_RAT	1	66.90	77.23	51.35	25.20	38.84	14.84
	10	70.25	75.35	72.45	29.71	38.11	33.14
HS90A_HUMAN	1	50.07	70.65	61.48	14.70	29.58	22.63
	10	48.90	74.17	71.58	13.85	41.69	31.03
LKHA4_HUMAN	1	94.39	92.48	93.49	59.29	62.98	67.54

LogAUC was computed with the following formula:

$$LogAUC_{\lambda} = \frac{1}{\log_{10}\frac{1}{\lambda}} \times \sum_{i}^{where\ x_i \geq \lambda} (\log_{10} x_{i+1} - \log_{10} x_i) \times \left(\frac{y_{i+1} - y_1}{2}\right)$$

Table 5. Enrichment factors and true positive percent at 5% decoys of ChemPLP, LID and GRIM in virtual screening challenge with the DUD-E and PubChem (in bold) dataset.

Uniprot ID	Number of protein structures	EF5%			EFmax	TP % at 5% decoys		
		ChemPLP	GRIM	LID		Chem PLP	GRIM	LID
ALDR_HUMAN	1	8.06	9.20	9.71	56.5	37.1	41.5	44.0
BACE1_HUMAN	1	10.1	6.69	10.5	63.9	45.2	31.4	49.4
CAH2_HUMAN	1	1.02	19.7	21.9	63.2	5.08	85.7	85.1
CDK2_HUMAN	1	3.38	4.27	9.53	58.7	16.6	21.3	45.5
ESR1_HUMAN	1	1.38	12.2	9.96	53.9	6.79	52.2	44.9
	**10**	**0.67**	**3.75**	**3.75**	**25.9**	**3.39**	**16.9**	**18.6**
GRIA2_RAT	1	3.81	9.88	1.27	74.8	18.3	46.8	6.33
	10	4.36	7.88	5.36	74.8	20.8	37.9	25.9
HS90A_HUMAN	1	0.91	4.86	3.07	55.1	4.55	23.8	15.9
	10	0.225	10.1	4.87	55.1	1.13	43.1	25.0
LKHA4_HUMAN	1	17.0	17.2	18.8	55.3	73.6	71.3	73.1

EF5% was computed with the following formula:

$$EF5\% = \frac{Actives_{5\%}/Compounds_{5\%}}{Actives_{all}/Compounds_{all}}$$

Table 6. Computation time of LID grid generation and scoring on the eight targets of the DUD-E dataset. Only active compounds are considered. The measures were done in triplicate. Std: standard deviation.

Uniprot ID	Number of poses	Number of interaction points in docked poses	Number of references	Number of interaction points in references	Grid generation time (s)		LID scoring time (s)	
					Mean	Std	Mean	Std
ALDR_HUMAN	2200	82425	41	4150	8.37	0.24	7.28	0.19
BACE1_HUMAN	4850	230795	227	24510	49.9	0.53	19.53	0.49
CAH2_HUMAN	8342	277604	156	8076	14.6	0.53	20.90	0.69
CDK2_HUMAN	7978	278549	195	14582	33.77	0.91	20.43	0.15
ESR1_HUMAN	6270	466733	37	4504	11.53	0.42	30.47	0.80
GRIA2_RAT	2970	87154	62	5028	11.77	0.21	5.97	0.15
HS90A_HUMAN	1250	57673	139	11440	25.27	0.15	6.04	0.29
LKHA4_HUMAN	2440	124538	26	2548	6.21	0.19	9.83	0.64

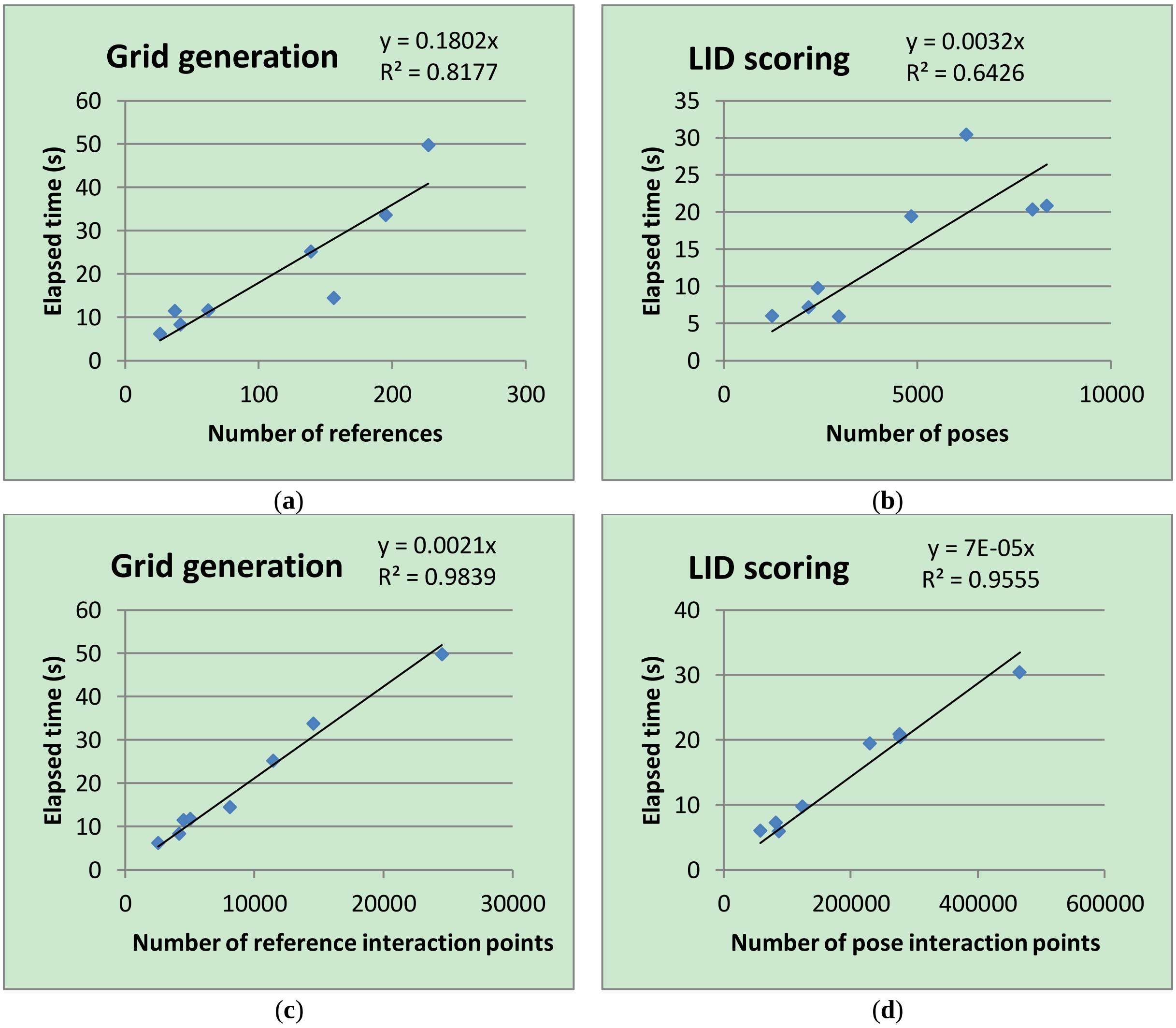

Figure 1. Correlation between the number of features and the computation time.

Table 7. Computation time of GRIM scoring on the eight targets of the DUD-E dataset. Only active compounds are considered. The measures were done in triplicate. Std: standard deviation.

Uniprot ID	Number of poses	Number of interaction points in poses	Number of reference	Number of interaction points in references	GRIM scoring time (s)	
					Mean	Std
ALDR_HUMAN	2200	82425	41	4150	810	30
BACE1_HUMAN	4850	230795	227	24510	11990	887
CAH2_HUMAN	8342	277604	156	8076	3931	88
CDK2_HUMAN	7978	278549	195	14582	7720	272
ESR1_HUMAN	6270	466733	37	4504	13430	540
GRIA2_RAT	2970	87154	62	5028	470	17
HS90A_HUMAN	1250	57673	139	11440	1830	79
LKHA4_HUMAN	2440	124538	26	2548	3410	121
